# Mass Absorption Coefficient of Tungsten and Tantalum, 1450 eV to 2350 eV: Experiment, Theory, and Application

**DOI:** 10.6028/jres.108.002

**Published:** 2003-02-01

**Authors:** Zachary H. Levine, Steven Grantham, Charles Tarrio, David J. Paterson, Ian McNulty, T. M. Levin, Alexei L. Ankudinov, John J. Rehr

**Affiliations:** National Institute of Standards and Technology, Gaithersburg, MD 20899-8410; Advanced Photon Source, Argonne National Laboratory, 9700 S. Cass Ave. Argonne, IL 60439; IBM Microelectronic Division, Essex Junction, VT 05452; Department of Physics, Box 351560 University of Washington, Seattle, WA 98195-1560

**Keywords:** Integrated circuit interconnect, M_3_ edge, M_4_ edge, M_5_ edge, mass absorption, microspectroscopy, tantalum, transmission, tungsten

## Abstract

The mass absorption coefficients of tungsten and tantalum were measured with soft x-ray photons from 1450 eV to 2350 eV using an undulator source. This region includes the M_3_, M_4_, and M_5_ absorption edges. X-ray absorption fine structure was calculated within a real-space multiple scattering formalism; the predicted structure was observed for tungsten and to a lesser degree tantalum as well. Separately, the effects of dynamic screening were observed as shown by an atomic calculation within the relativistic time-dependent local-density approximation. Dynamic screening effects influence the spectra at the 25 % level and are observed for both tungsten and tantalum. We applied these results to characterize spatially-resolved spectra of a tungsten integrated circuit interconnect obtained using a scanning transmission x-ray microscope. The results indicate tungsten fiducial markers were deposited into silica trenches with a depths of 50 % and 60 % of the markers’ heights.

## 1. Introduction

The technological challenge of obtaining two and three dimensional x-ray images of integrated circuit interconnects [16,17,18,28] spurred some of us to measure the spectrum of tungsten [15], a key material in integrated circuit interconnects, in the region of its M_4_ and M_5_ edges. The hope is that improved knowledge of the spectra will enable identification of these materials *in situ*. Our need for this information made us aware that fundamental understanding of M-edge heavy metal spectra was not yet fully quantitative either experimentally or theoretically [5]. While the observation of M_3_, M_4_, and M_5_ edges was reported many years ago [8,31], only the energies were given, not the mass absorption coefficient. Prior measurements of the mass absorption coefficient of tungsten [13,21] and tantalum [9] in this spectral region are limited.

In this work, we repeated the tungsten experiment with improved statistics, extended the energy range to 1450 eV to 2350 eV from 1600 eV to 2100 eV which permitted observation of the M_3_ edges, and improved both our absolute and relative accuracies. The work also includes tantalum, a material used for the liners of integrated circuit interconnects to provide a diffusion barrier between copper and silica. Since Ta and W have adjacent atomic numbers, namely 73 and 74, studying them together helps identify trends and systematic uncertainties. In the previous experiment [15], our results indicated that tungsten was more absorbing than had been predicted theoretically and, to a lesser extent, than had been observed with line sources [13,21]. A key feature of the present experiment is an absolute determination of the mass thickness of the film using both a high precision balance as well as x-ray reflectivity with a Cu K*_α_* source.

In accordance with our original motivation, the tungsten spectra were used in a preliminary microspectroscopy study of an integrated circuit interconnect sample. Tungsten lines were clearly identified and their thicknesses measured in a matrix of silica.

## 2. Experiment

### 2.1 Sample Preparation and Characterization

Samples were prepared using direct current (DC) magnetron sputtering. The base pressure of the vacuum chamber was 1 *µ*Pa (8 nTorr). Argon gas of 99.999 % purity flowed into the system at 70 cm^3^/min (if at standard temperature and pressure), leading to an Ar pressure of 0.25 Pa (2 mTorr). The sputtering power was 80 W, and targets were of 99.95 % purity. Five test depositions of tungsten and two of tantalum were made on float-glass substrates. Substrates were located 100 mm from the source. These test films were measured using grazing-incidence x-ray reflectivity with Cu K*_α_* radiation (*λ* = 0.154 nm), and the data were fit using the Fresnel equations [12]. The thicknesses were determined to within ± 0.5 %, and the deposition rate was constant to within ± 2 %. (All uncertainties given in this paper are expanded, combined uncertainties with 95 % confidence intervals.) Using the Henke et al. [14] values of the atomic scattering factors, the fits yielded film densities of 0.97 ± 0.03 and 0.97 ± 0.03 of bulk density for the tantalum and tungsten films, respectively. Two additional test depositions of Ta were made on 25 mm diameter Si wafers that were weighed before and after deposition. The density measured in this fashion was determined to be 0.97 ± 0.03 of the bulk tantalum density. The result represents an independent check on the determination of the density using x rays. This measurement included fractional uncertainties in the area (0.02), mass (0.015), and deposition rate (0.02).

The films used in the measurements were deposited on 1 mm^2^ area, 100 nm thick Si_3_N_4_ membranes supported by Si frames, and were 50 nm ± 1 nm and 150 nm ± 3 nm thick for both tungsten and tantalum. Because the metal films are highly stressed when grown, some wrinkling occurred. The samples were examined by reflecting a laser from the surface, and the maximum out-of-plane slope of the surface was determined to be 5°. The path through the wrinkled film thickness is up to 1/cos(*θ*) ≈ 1.004 times the path through a flat film; this factor is neglected. All samples were stored in dessicators for the period between preparation and measurement; the films were exposed to atmosphere for about one hour for examination and mounting, plus up to two days at the beamline during which the time of exposure to air for all samples was identical.

### 2.2 Synchrotron Measurement

The measurements were made at undulator beamline 2-ID-B of the Advanced Photon Source (APS). [24] The undulator beam is monochromatized by a water-cooled spherical grating monochromator with a constant deviation angle of 4.5°. The monochromator uses a rhodium-coated grating with ion-etched laminar grooves and has adjustable entrance and exit slits. Harmonics of the undulator fundamental energy are effectively suppressed above 2.8 keV by two rhodium-coated mirrors operating in tandem at grazing incidence angles of 1.25°. Including the diffraction efficiency of the grating, harmonic content is conservatively estimated to be below 1 %. The flux transmitted by the sample was detected with a calibrated silicon photodiode with a 180 nm Ti filter. These photodiodes have excellent response uniformity, very stable quantum efficiency, and NIST-traceable responsivity over the 100 eV to 4000 eV photon energy range [11]. Due to the presence of silicon in the beamline optics and the photodiode, a pronounced Si K edge was evident in the data. We added a constant value of 3 eV to the nominal calibration of the monochromator to set the known position (1839 eV) of this edge.

The x-ray beam passed through an aperture with 100 *µ* m diameter. Both the samples and the photodiode were in air. The thinned part of the samples was 1 mm square. Hence, it was relatively straightforward to align the beam to the samples. The incident photon energy was scanned in uniform energy steps in three intervals for each material, as shown in Table 1. The step sizes and calculated monochromator resolution are also shown in the table. (The region 1753 eV to 1803 eV was inadvertently omitted for W.) The middle interval for W was performed with higher resolution and smaller step size than the others to observe its XAFS spectra. Data were accumulated for 1 s in every observation; the time required to change energies was also approximately 1 s. The energy was scanned through each energy interval several times, as shown in Table 1. Multiple scans in each interval helped average over the noise which was dominated by fluctuations in the beam intensity. Also, the energy scale was subject to a jitter of approximately 2 eV when runs were repeated; multiple runs allowed this noise source to be averaged as well.

**Fig. 1:**
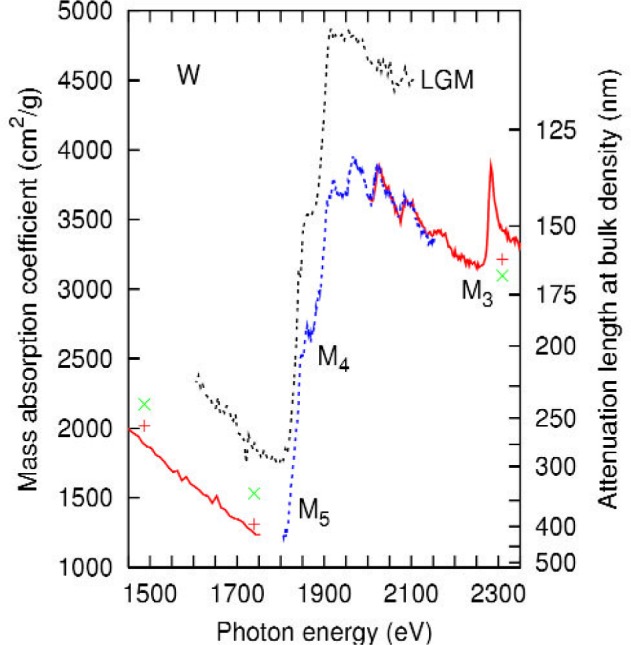
Present experimental results for the mass absorption coefficient of tungsten in thin solid lines (lower and higher energy intervals) and dotted lines (middle energy interval) compared to previous experimental results. See Table 1 for descriptions of the energy intervals. Data from Ref. [15] are labeled LGM. Line source data from Ref. [13] (green ×) and Ref. [21] (red +). The M_3_, M_4_, and M_5_ edges were observed as well as XAFS above the M_4_ edge and between the M_5_ and M_4_ edges. (Colors refer to on-line version only.)

The transmission coefficient through the interior tungsten or tantalum [10,15] was calculated by forming the ratio

$$T=\frac{I_{\text{thick}}-I_{\text{back}}}{I_{\text{thin}}-I_{\text{back}}}$$ (1)

where *I*_thick_ and *I*_thin_ were the observed intensities with the thick and thin samples in place respectively, and *I*_back_ was the observed signal with the beam off. *I*_back_ was at most 10 % of *I*_thick_. This method has the advantage of correcting for the beamline, air path, and detector efficiencies; in particular the experiment is relatively immune to surface effects. Our longest duration scan, tungsten from 1803 eV to 2153 eV with 1 eV steps, was obtained in unattended operation. In this case, the beam intensity varied by a factor of 3.4 in 2.2 h, i.e., by about a factor of 1.2 from scan to scan. The variation was a smooth, monotonic decrease. The beam intensity was normalized by summing the recorded signal for the thin samples and fitting the log of the intensity as a function of time with splines. The beam intensity for the thick samples was estimated using this fit. The excellent agreement between the data obtained with and without the extra normalization procedure between 2000 eV and 2150 eV (shown in Fig. 1) suggests the procedure did not introduce artifacts. To a great extent, observations were made in the order thin-thick-thick-thin to minimize the effects of variations in the beam intensity. However, data sets were discarded when anomalies indicated incomplete noise reduction.

The mass absorption coefficient *η* is determined from Beer’s Law

$$T=e^{-\eta \rho t},$$

where *ρ* is the density and *t* is the sample thickness (or, thickness difference in our case). As discussed above, we measured the density *ρ* to be 0.97 ± 0.03 and 0.97 ± 0.03 times the bulk densities of 19.3 g/cm^3^ and 16.6 g/cm^3^ for tungsten and tantalum, respectively. For the theory, it was necessary to convert calculated cross sections using the atomic weights 183.84 u for tungsten and 180.95 u for tantalum, where 1 u = 1.66053873 × 10^−24^ g. At bulk density, a value typical in this experiment for the mass absorption coefficient 1000 cm^2^/g corresponds to an attenuation length of 518 nm for tungsten and 602 nm for tantalum. Hence the attenuation lengths of these x-ray energies are well matched to the scale of integrated circuit interconnects.

The uncertainty due to the measurement other than the characteristics of the sample, e.g., fluctuations in the beam intensity and in the relation between the nominal and actual energy (2 eV) lead to an uncertainty of 100 cm^2^/g in any given data point, in addition to the 3 % uncertainty in the overall scale.

## 3. Mass Absorption Coefficient of Tungsten

In Fig. 1, our data are compared to experimental mass absorption data on tungsten in this spectral region from line source data [13,21] and previous synchrotron data [15]. The agreement with the data of Ref. [21] is within joint uncertainties. The present data lie outside of the 95 % confidence interval of data we have given previously [15]. The origin of this difference is unknown although our conjecture is that the thickness of the samples in the previous experiment was determined inaccurately. The principal features of the spectrum are the M_5_ and M_4_ edges, nominally [4] at 1809 eV and 1872 eV, and the M_3_ edge at 2281 eV.

**Fig. 2:**
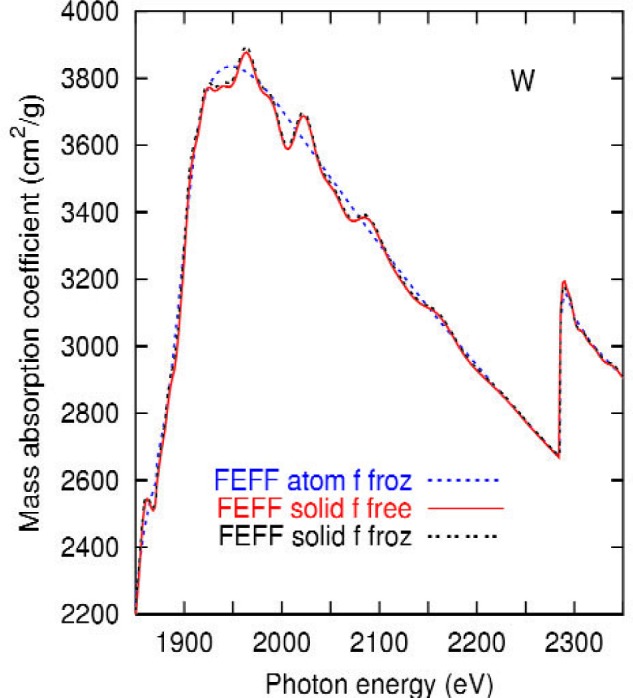
Results of the real-space multiple scattering calculating treating the *f*-electrons as active valence electrons and as frozen core elements. Also shown is the effective-medium atomic result. The result does not depend on whether the *f*-electrons are frozen. This justifies freezing them in the case of tantalum which led to numerical difficulties for the feff program, leading to the creation of a new version of the code with frozen *f*-electrons. The graph is cropped to emphasize the XAFS region.

As in Ref. [15], we performed a real-space multiple scattering calculation of the cross section known as feff 8.1 [2,3]. Preparation of the input to this code was simplified by the use of the atoms code [25]. These calculations and their relation to x-ray absorption structure (XAFS) have been reviewed recently [26]. The calculation reported here differs somewhat from that given in Ref. [15] due to improved numerical procedures and the inclusion of a Debye-Waller factor for 300 K. Figure 2 illustrates that the oscillations are imposed on the single effective-medium [27] atomic calculation by the surrounding atoms. Also shown is the negligible difference in treating the *f* electrons as core or valence electrons.

**Fig. 3:**
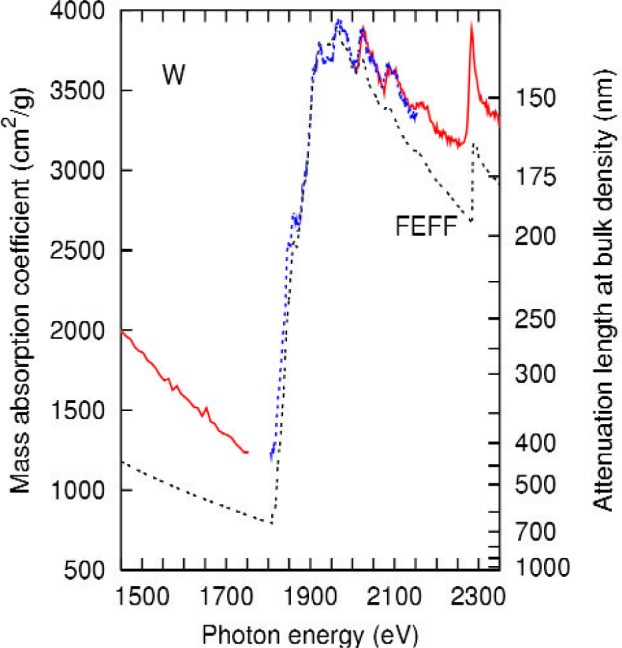
Experimental results (labeled as in Fig. 1 compared to real-space multiple scattering calculation for tungsten (labeled feff). The experimental data given alternately as solid and dotted lines represent data taken in separate passes of the spectrometer. The difference between the two runs helps to estimate the experimental uncertainty. Note that the XAFS predicted by the theory, including one small peak between the M_5_ and M_4_ edges, is seen in the experiment, but the overall shape is not in full agreement.

**Fig. 4:**
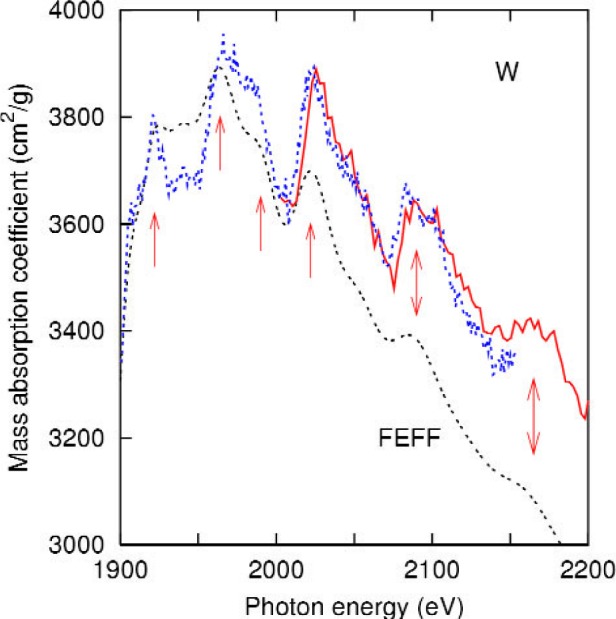
Expanded view of Fig. 3 showing the x-ray absorption fine structure in both theory and experiment. The arrows indicate peaks which are in agreement between the theory and the experiment.

**Fig. 5:**
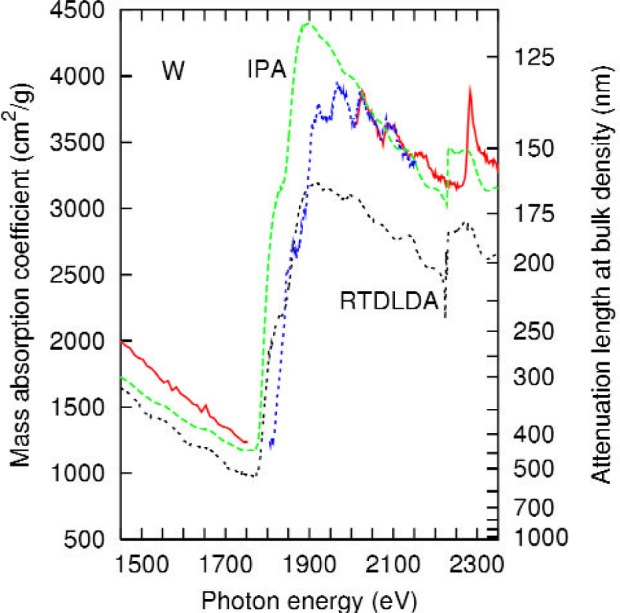
Experimental results (labeled as in Fig. 1) compared to an atomic calculation with (RTDLDA) and without (IPA) dynamic screening. Note that the atomic calculation without dynamic screening resembles the feff real-space multiple scattering calculation.

A comparison of the present data with the results of feff is shown in Fig. 3. The calculation accounts for the main peaks and the oscillations between 1900 eV and 2150 eV, shown in more detail in Fig. 4. There are six peak positions in agreement at least 30 eV above the tungsten M_4_ edge at 1872 eV. feff calculations of the XAFS of metals are generally valid 30 eV or more above an edge [26], but the Hedin-Lundqvist plasmonpole self energy used tends to overestimate losses at low energies leading to theoretical oscillations which underestimate the experiment. Consideration of Fig. 3 indicates that the six peaks between the M_4_ and M_3_ edges as well as one small peak between the M_5_ and M_4_ edges are XAFS, i.e., induced by neighboring atoms, but the edges themselves—M_5_, M_4_, and M_3_—are atomic phenomena. However, certain of the large scale features of the spectrum are not perfectly described. In particular, the pre-edge regime has the wrong slope, the peak associated with the M_5,4_ transition is a too large (measured from the base) and the mass absorption coefficient decreases somewhat too rapidly past the M_4_ peak. Interestingly, these large-scale defects are not present in an atomic calculation including dynamic screening known as the Relativistic Time-Dependent Local-Density Approximation (RTDLDA) [20], presented in Fig. 5. The slope of the pre-edge structure is well accounted for, (see Table 2), as is the general character of the M_5,4_ transition and the spectrum up through the M_3_ edge. A version of this calculation known as the Independent Particle Approximation (IPA) is also given; this calculation uses the same wave functions but uses the conventional dipole operator for the transition matrix elements rather than the screened field [32]. We note that the IPA shares some of the flaws of the feff result noted above. These results suggest that combining the two approaches would result in a more realistic calculation.

Recently, dynamic screening has been proposed as a necessary physical feature to understand the branching ratios in the L_2,3_-shells of the transition metals [1]. Specifically, dynamic screening can either increase or decrease this ratio by up to a factor of 2 from the ratio depending on the atomic number. As seen in Table 2 the statistical ratio is not within the 95 % confidence interval for the present experiment. The RTDLDA calculation, including dynamic screening, accounts for the ratio.

## 4. Mass Absorption Coefficient of Tantalum

**Fig. 6:**
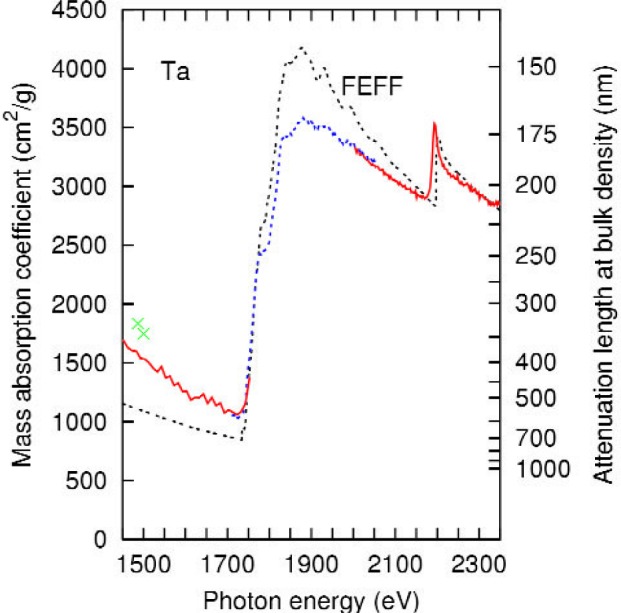
Experimental results for tantalum (labeled as in Fig. 1) compared to the real-space multiple scattering calculation labeled feff. Also shown is line source data from Ref. [9].

The experimental results are shown in Fig. 6 along with those of the feff calculation and previous experimental results [9]. It is not surprising that the results for tantalum with atomic number *Z* = 73 are similar to tungsten with *Z* = 74. Again, the M_5_, M_4_, and M_3_ edges nominally at 1735 eV, 1793 eV, and 2194 eV are dominant structures in both the experiment and the theory. Four XAFS peaks above the M_4_ edge are also visible. These are not as prominent in the experiment for Ta as for W because the theory predicts the structures to be a factor of two smaller and because the monochromator resolution was somewhat worse for the tantalum measurement, as listed in Table 1.

Reference [9] reports a measurement of the mass absorption coefficient of thin film Ta samples at 1486.7 eV (Al K*_α_*) the value is 1835 ± 55 cm^2^/g and at 1500 eV, 1748 ± 52 cm^2^/g. Our values, after linear interpolation, are 1578 ± 147 cm^2^/g and 1534 ± 146 cm^2^/g, at these energies, respectively. This represents a modest disagreement. The principal differences between the experiment are: our use of a synchrotron source vs a tube source (both line and continuum) for Ref. [9], sputtered films vs evaporated films for Ref. [9], and our use of pairs of films to eliminate surface effects vs the use of single films in Ref. [9].

For tantalum, the existing feff 8.1 code required the *f* electrons to be treated as valence electrons, yet produced an unphysical partial occupation of the *f* band with a resultant white line spectrum. The code was extended to permit these electrons to be frozen. As shown in Fig. 2, this approximation has a negligible effect on tungsten; presumably the same is true for tantalum. In any event, the tantalum calculations are presented with the *f* electrons treated as core.

**Fig. 7:**
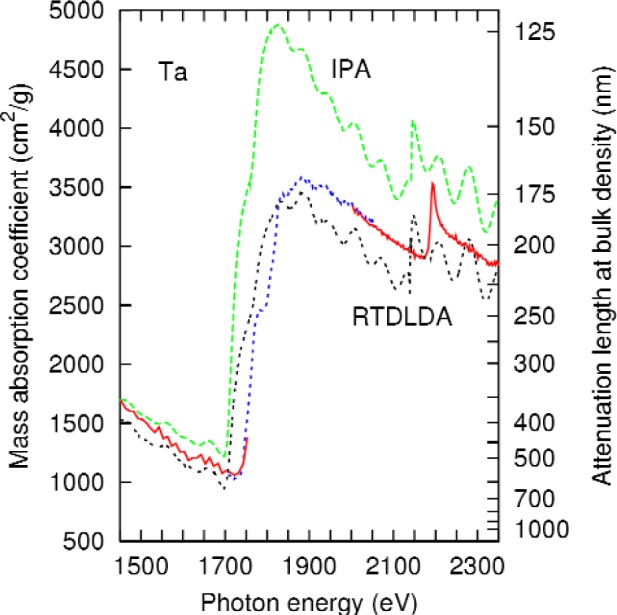
Experimental results (labeled as in Fig. 1) compared to an atomic calculation with (RTDLDA) and without (IPA) dynamic screening. See caption to Fig. 5.

As for the case of tungsten, the pre-M_5_ edge slope in the calculation is insufficiently negative, the post-M_4_ slope is too negative, and the peak height of the combined M_5_, M_4_ transition is too large. As for tungsten, an RTDLDA calculation was performed. The results are presented in Fig. 7. Again, many of the conclusions from the tungsten calculation follow: the dynamically screened results give a better overall account of the main peak height and the slope above the M_4_ edge. Table 2, indicates that dynamic screening aids in the description of the pre-M_5_-edge behavior, as well as the branching ratio *τ*. The similarity of the feff results and the IPA are notable, as they were for tungsten. On balance, the tantalum results reinforce the conclusion that both dynamic screening and multiple-scattering effects are important for the understanding of these spectra.

## 5. Microspectroscopy of an Integrated Circuit Interconnect

**Fig. 8:**
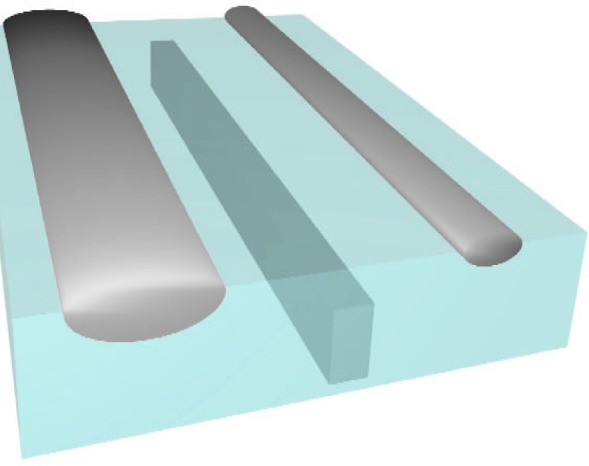
Sketch of the interconnect sample using width and depth information obtained from x-ray microscopy and microspectroscopy, respectively. Two tungsten FIB marker are placed in shallow trenches of silica which was presumably removed as the markers were created. A tungsten interconnect line is enclosed in silica. The spectra of Fig. 9 were taken from the centers of the metal lines shown here; from left to right, these are “FIB-1”, “interconnect”, and “FIB-2”.

An integrated circuit sample was prepared for x-ray transmission measurements by removing the back of the sample through the use of a focused ion beam (FIB). Fiducial markers consisting of a thick and several thin tungsten bars were added to the surface of the circuit. Some of these markers surround a tungsten interconnect which was located in the sample. A sketch of the sample is shown in Fig. 8.

Microspectra were obtained at 11 photon energies using the scanning transmission x-ray microscope at the 2-ID-B beamline [24]. The microscope had a 98 *µ* m diameter Fresnel zone plate with an outer zone width of 40 nm and integrated gold central stop with a 40 *µ* m diameter. Images of 201 × 15 pixels with a step size of 40 nm × 80 nm were obtained for each energy. The lateral scale is uncertain by ± 7 %. The images were cropped to 140 × 15 pixels to contain one large FIB marker 1070 nm ± 70 nm the buried tungsten interconnect 1510 nm ± 110 nm away (center to center) with a width of 320 nm ± 20 nm, one small FIB marker centered 1140 nm ± 80 nm from the center of the interconnect with a width of 520 nm ± 40 nm, and silica at each edge. The individual rows of each image acquired at a given photon energy were mutually aligned in software after the data were acquired. The images could be assumed to be periodic in *x* because both sides had only silica for a considerable region near their edges. This enabled the correlations to be found in the Fourier domain, and noninteger alignment shifts were chosen to yield maximum correlations. After alignment, the pixels in each column (i.e., points corresponding to the same distance from the edge of each metal line) were summed. These values—effectively line scans—at 11 photon energies were then aligned to each other using the same algorithm. The transmitted intensity was adjusted for dark counts then normalized (by division) for each photon energy to the transmission through a region of silica 32 pixels wide (1280 nm ± 90 nm) far from any metal line using Eq. (1).

**Fig. 9:**
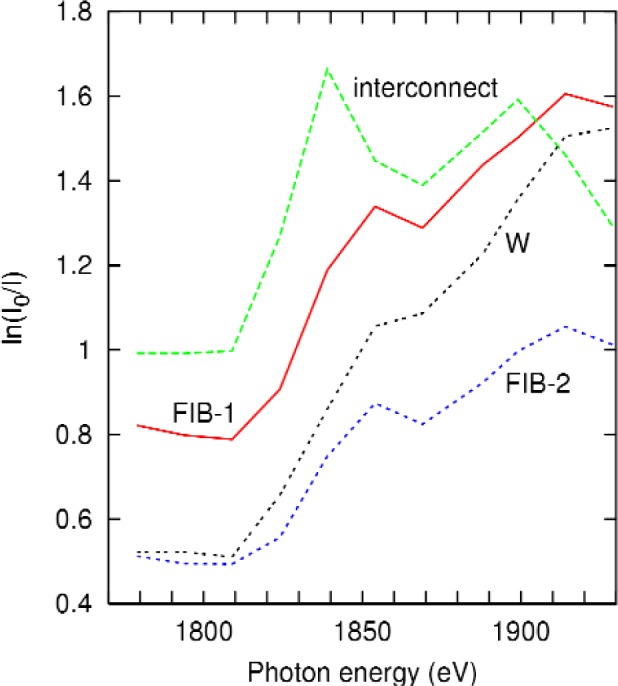
Spectra of tungsten (scaled to 220 nm at bulk density), and spectra obtained at three locations in the sample: from a large tungsten FIB marker (FIB-1), a small tungsten FIB marker (FIB-2), and a buried tungsten interconnect.

The spectra at the centers of the three features are shown in Fig. 9 along with the measured tungsten spectrum of Fig. 1. For 1779 eV and 1794 eV, the present results were interpolated with the aid of the earlier measurements [15]. The spectra of the tungsten FIB markers were proportional to each other with a ratio of 0.64 ± 0.04. However, they were merely correlated with the measured tungsten spectra. When the FIB lines were deposited, they were sufficiently energetic to create a trench in the silica. Given our normalization, such a trench represented a negative amount of silica in the beam. The tungsten interconnect, which was created with the standard multilayer lithographic process has, assuming no voiding, a given amount of tungsten and an equal amount of silica removed. Hence, we fitted the spectra of the large and the small FIB markers as linear combinations [19] of 135 nm of W plus 0.428 times the interconnect’s spectrum and 100 nm of W plus 0.235 times the interconnect’s spectrum, respectively. Root mean square (rms) residuals were less than 2 % of the rms value of the original spectra in each case. This small residual is the strongest evidence for the model shown in Fig. 8. To obtain the thickness of the interconnect, the silica spectrum at our measured energies below the silicon K edge was estimated using x-ray absorption tables [14]. In this regime, the rms value of the silica absorption spectrum is 6 % of the corresponding measured tungsten spectrum. The difference spectrum of tungsten minus silica is formed with uncertainties in the correction of 1 % of the total. The interconnect is found to be 450 nm ± 40 nm thick. Among the four energies below the silicon K edge, the measured spectrum of the interconnect is fit to the difference spectrum with a 1 % residual.

The net result is an estimate of 330 nm ± 30 nm of W with 190 nm ± 20 nm of silica removed for the larger FIB marker and 200 nm ± 20 nm of W with 100 nm ± 10 nm of silica removed for the smaller one. The uncertainty estimate is based on the Type A uncertainties of 2 %, Type B uncertainties of 5 % and an uncertainty of 6 % associated with the absolute value of the W spectrum. A sketch which is consistent with these dimensions and those obtained in the horizontal direction through our x-ray images at any single frequency is shown in Fig. 8.

## 6. Summary and Conclusions

The mass absorption coefficients of tantalum and tungsten thin films were measured over the energy range 1450 eV to 2350 eV using synchrotron radiation. As shown below, the new values for the tungsten mass absorption coefficient are in better agreement with the bulk of previous theory and experiment than are the values of Ref. [15]. The new measurement was sufficiently precise to allow determination of the x-ray absorption fine structure (XAFS). Moreover, evidence for collective effects in the spectrum was found by consideration of a calculation performed within the Relativistic Time-Dependent Local-Density Approximation (RTDLDA) [20]. In addition, the values were calculated using a real-space multiple scattering code [2,3]. Including uncertainties of the area and the thickness, the absolute scale was determined to ± 3 %, compared to ± 27 % in Ref. [15]. There is also an additional uncertainty of ± 100 cm^2^/g due to effects associated with the beamline. Agreement with an experiment for the mass absorption coefficient of tungsten obtained from line sources [21] was obtained to within joint uncertainties.

The energy range includes the M_3_, M_4_, and M_5_ edges for both materials. The substantial widths of the M_3_, M_4_ edges measured previously [15] were confirmed here and extended to Ta; they are accounted for within the real-space multiple scattering calculation. In addition, the relatively narrow M_3_ edge was observed similar to the L_3_ edge observed in tantalum many years ago [29] and is also in agreement with the prediction of the real-space multiple scattering code presented herein. For tungsten, about five full cycles of the x-ray absorption fine structure were observed and found to be in excellent agreement with the predictions of real-space multiple scattering with the minor exception of the shape of the second peak. Similar structures were observed in tantalum, although less definitively.

In contrast, the real-space multiple scattering code (feff) did not predict the slope of the mass-absorption coefficient below the threshold, overestimated the height of the main peak, and predicted a steeper fall-off in the cross section above the M_4_ edges for both tungsten and tantalum than was observed. However, the atomic code was instructive: without dynamic screening (IPA), the errors were qualitatively similar to that of the multiple-scattering (feff) calculation. Including dynamic screening (RTDLDA) led to substantial improvements in the ability to account for the slope below the M_5_ edges, the height of the main peak (just above the M_4_ edges) and the slope. These changes were about a part in three. Although it has been known for some time that dynamic screening has a large effect on photoemission from valence levels [32], it is a surprise to see a 30 % effect given that the index of refraction of tungsten at, e.g., 1860 eV [22] is quoted as 0.999810 + *i* 0.000344, i.e., nearly unity.

Can dynamic screening and multiple-scattering effects be combined? Almost certainly. Results from molecular calculations in BaC_60_ [30] and BaO_2_ [7] show the giant dipole resonance of atomic barium has x-ray absorption fine structure due to modulation of the molecular environment. One promising way to implement this would be to use atomic RTDLDA matrix elements and the XAFS from feff [1].

Our experimental results on tungsten were used to confirm the presence and estimate the thickness of tungsten features in an integrated circuit interconnect using an x-ray transmission microscope. Armed with this more complete understanding of the absorption coefficients of W and Ta, one may now identify buried interconnects *in situ* and measure their thicknesses quantitatively.

## Figures and Tables

**Table 1:** Calculated monochromator resolution Δ*E* and other parameters for various runs [23]. *N* is the number of observations of a pair of samples for each energy interval. The monochromator slit settings were changed in different intervals; within each interval, the resolution changes monotonically. All energies are given in electron volts. *ħω* is the energy of the x-ray photon at the lower and upper ends of each interval

	*N*	Step size	*ħω*	Δ*E*	*ħω*	Δ*E*
Ta	6	10.0	1453	2.5	1753	3.5
Ta	7	2.5	1703	3.3	2053	4.2
Ta	8	2.5	2003	4.0	2353	5.5
W	9	10.0	1453	2.5	1753	3.5
W	7	1.0	1803	2.6	2153	3.7
W	8	2.5	2003	4.0	2353	5.5

**Table 2:** Two parameters related to the mass absorption coefficient *η* of W: (1) the experiment *α* relating the mass absorption coefficient and the energy via *η* ∝^−^*^α^* below the threshold and (2) the branching ratio *τ* of the M_5_ contribution to the M_4_ contribution of the mass absorption coefficient of W. “General values” refers to averages over many elements from experiments performed in the early days of quantum mechanics. “Statistical ratio” is the ratio of the number of electrons in the 3*d*_5/2_ and 3*d*_3/2_ orbitals

	W		Ta	
	*α*	*τ*	*α*	*τ*
Expt. [13]	2.23			
Expt. [21]	2.74			
Present IPA [20]	2.01	1.59	2.02	1.71
Present RTDLDA [20]	2.76	1.23	2.60	1.40
Present feff [2,3]	1.80	1.51	1.75	1.40
Expt. [15]	2.74 ± 0.81	1.30 ± 0.30		
Present expt.	2.56 ± 0.25	1.26 ± 0.20	2.71 ± 0.30	1.24 ± 0.20
General values [6]	2.5–3.0		2.5–3.0	
Statistical ratio		1.5		1.5
